# Germline variants in patients developing second malignant neoplasms after therapy for pediatric acute lymphoblastic leukemia—a case-control study

**DOI:** 10.1038/s41375-024-02173-2

**Published:** 2024-02-27

**Authors:** Stefanie V. Junk, Alisa Förster, Gunnar Schmidt, Martin Zimmermann, Birthe Fedders, Bernd Haermeyer, Anke K. Bergmann, Anja Möricke, Gunnar Cario, Bernd Auber, Martin Schrappe, Christian P. Kratz, Martin Stanulla

**Affiliations:** 1 Pediatric Hematology and Oncology, Hannover Medical School, Hannover, Germany; 2 Department of Pediatric Oncology, Hematology and Clinical Immunology, Medical Faculty, Heinrich Heine University Düsseldorf, Düsseldorf, Germany; 3 Institute of Human Genetics, Hannover Medical School, Hannover, Germany; 4 Department of Pediatrics, University Hospital Schleswig-Holstein, Kiel, Germany

**Keywords:** Genetics research, Acute lymphocytic leukaemia, Cancer prevention

## To the Editor:

Today, most children treated for acute lymphoblastic leukemia (ALL) can be cured by the application of intensive combination chemotherapy regimens [1, 2]. However, within 20 years, up to 10% develop a second malignant neoplasm (SMN) with cure rates often being dismal [3, 4]. Therefore, strategies are needed for an early identification of patients at risk for SMN development, an improved understanding of the underlying pathobiology and, ideally, the development of preventive actions. In this study, we analyzed genetic variation in 159 selected cancer predisposition genes (CPG) to determine their potential association with SMN.

Patients included were 1–22 years of age at diagnosis of ALL and enrolled in the German part of the European AIEOP-BFM ALL 2000 multicenter clinical trial for frontline treatment of pediatric ALL [5]. Median follow-up for the entire patient group was 14.8 (4.6–19.2) years as of November, 2020. Treatment details were described previously [6]. We identified 75 SMN patients with sufficient DNA available, representing 94% of all 80 SMN cases observed in the entire AIEOP-BFM ALL 2000 study population up to this follow-up date. In a matched case-control approach, we analyzed germline DNA obtained from remission bone marrow samples of 223 individuals (75 cases, 148 controls); see Supplementary Methods section for sequencing details and Supplementary Table 1 for clinical background information. Matching criteria were sex, age at diagnosis of ALL, immunophenotype, and treatment. Candidate genes included genes based on own work and/or genes recommended by Byrjalsen et al. 2021; [7] variant pathogenicity was assessed according to standard interpretation guidelines (see Supplementary Table 2 for details). Statistical analyses and plotting were conducted with SAS (SAS-PC, Version 9.1, Cary, NC: SAS Institute Inc.), SPSS (IBM Deutschland GmbH, Ehningen, Germany) or R (4.0.5), and Rstudio (1.0.153); see Supplementary Methods section.

**Fig. 1:**
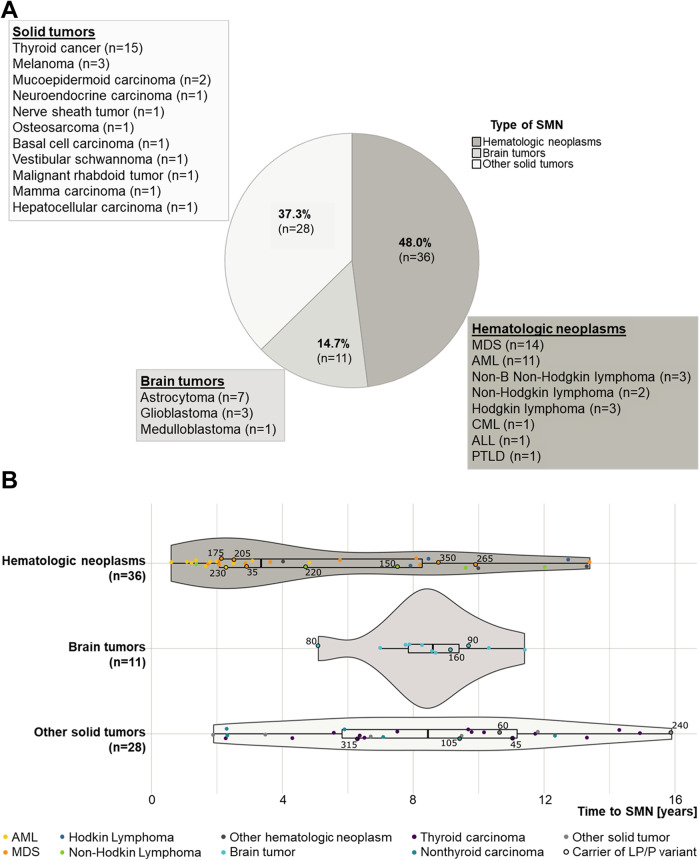
Characteristics of second malignant neoplasms (SMN) in the 75 included patients from trial AIEOP-BFM ALL 2000. **A** Entities of SMN; (**B**) time from diagnosis of the acute lymphoblastic leukemia (ALL) to SMN development; median time to SMN was 3.36 ± 3.98 (0.57–13.37) years for patients with hematologic SMN, 8.61 ± 1.69 (5.12–11.43) years for patients with second brain tumors, and 8.45 ± 4.04 (1.92–15.91) years for patients with other second solid tumors. Black-bordered dots represent patients with a (likely) pathogenic variant (LP/P) in one of the candidate genes; the adjacent numbers correspond to the respective patient IDs (for further details, compare Table 1 and Supplementary Tables 3, 4). Abbreviations: MDS myelodysplastic syndrome, AML acute myeloid leukemia, CML chronic myeloid leukemia, PTLD post-transplant lymphoproliferative disorder.

Overall we observed 31 conspicuous findings in 22 genes: 17 (6 likely pathogenic and 11 pathogenic) in 16 (21%) cases with ALL and subsequent SMN and 14 variants (9 likely pathogenic and 5 pathogenic) were detected in 14 (9%) control patients with ALL; for details see Table 1 and Fig. 1. While some variants were found in patients from both groups (e.g., rs28909982 in *CHEK2*, rs113993993 in *SBDS* and rs764079291 in *NF1*), certain genes were exclusively altered in SMN patients (e.g., *ATM, TP53, A2ML1, MSH6 and PMS2*).

**Table 1:** (Likely) pathogenic variants determined in patients with acute lymphoblastic leukemia with or without second malignancies.

Patients with ALL and subsequent SMN (*n* = 75)					Gene^d^	Patients with ALL (*n* = 148)			
GRCh37 (hg19)^a^	Consequence	Entity^b^	Variant ID	Class^c^		GRCh37 (hg19)^a^	Consequence	Variant ID	Class^c^
11-108188099-G-T	NM_000051.4(*ATM*):c.6199-1G > T p.?^e^	A	rs1591788932	4	***ATM***				
11-108115654-C-T	NM_000051.4(*ATM*):c.802C > T p.(Gln268*)^f^	A	rs557012154	5					
					***RECQL4***	8-145738522-C-G	NM_004260.4(*RECQL4*):c.2464-1G > C p.?	rs398124117	4
					***CDH1***^**h**^	16-68862112-A-T	NM_004360.5(*CDH1*):c.2200A > T p.(Arg734*)	-	4
					***BRCA1***^**h**^	17-41197784-G-A	NM_007294.4(*BRCA1*):c.5503C > T p.(Arg1835*)	rs41293465	5
13-32907428--A	NM_000059.4(*BRCA2*):c.1813dup p.(Ile605Asnfs*11)	H	rs80359306	5	***BRCA2***				
					***FANCD2***	3-10130147-C-T	NM_033084.6(*FANCD2*):c.3481C > T p.(Gln1161*)	rs369022159	4
						3-10127560-C-T	NM_033084.6(*FANCD2*):c.3289C > T p.(Arg1097*)	-	4
17-7577539-G-A	NM_000546.6(*TP53*):c.742C > T p.(Arg248Trp)^f^	H	rs121912651	5	***TP53***^**h**^				
22-29121326-T-C	NM_007194.4(*CHEK2*):c.349A > G p.(Arg117Gly)^f^	H	rs28909982	4	***CHEK2***^**h**^	22-29121326-T-C	NM_007194.4(*CHEK2*):c.349A > G p.(Arg117Gly)	rs28909982	4
2-48030647--C	NM_000179.3(*MSH6*):c.3261dup p.(Phe1088Leufs*5)	S	rs267608078	5	***MSH6***^**h**^				
7-6026988--AC	NM_000535.7(*PMS2*):c.1408delins47 p.(Pro470Valfs*3)^e, g^	A	-	5	***PMS2***^**h**^				
17-29528489-C-T	NM_000267.3(*NF1*):c.1246C > T p.(Arg416*)	A	rs764079291	5	***NF1***^**h**^	17-29528489-C-T	NM_000267.3(*NF1*):c.1246C > T p.(Arg416*)^i^	rs764079291	5
					***NBN***	8-90983445-GTTTT--	NM_002485.5(*NBN*):c.657_661delACAAA p.(Lys219Asnfs*16)^g^	rs587776650	5
12-9007428-G-A	NM_144670.6(*A2ML1*):c.2764+1G > A p.(?)	S	rs773034576	4	***A2ML1***^**h**^				
12-25398285-C-G	NM_004985.5(*KRAS*):c.34G > Cp.(Gly12Arg)^j^	H	rs121913530	5	***KRAS***^**h**^				
22-21344765-G-A	NM_006767.4(*LZTR1*):c.742G > A p.(Gly248Arg)^f^	H	rs869320686	5	***LZTR1***^**h**^	22-21350154-C-T	NM_006767.4(*LZTR1*):c.2062C > T p.(Arg688Cys)	rs587777178	4
22-21342297-AG--	NM_006767.4(*LZTR1*):c.401-2_401-1del p.?	H	rs769200796	4					
					***PTPN11***^**h**^	12-112888157-A-G	NM_002834.5(*PTPN11*):c.173A > G p.(Asn58Ser)	rs751437780	4
16-3832811-G-A	NM_004380.3(*CREBBP*):c.1447C > T p.(Arg483*)	S	rs1555484797	5	***CREBBP***^**h**^				
7-66459273-T-A	NM_016038.4(*SBDS*):c.184A > T p.(Lys62*)	H	rs120074160	5	***SBDS***	7-66459197-A-G	NM_016038.4(*SBDS*):c.258 + 2T > C p.?	rs113993993	5
7-66459197-A-G	NM_016038.4(*SBDS*):c.258+2T > C p.?	S	rs113993993	5		7-66459197-A-G	NM_016038.4(*SBDS*):c.258 + 2T > C p.?	rs113993993	5
5-176638471-C-G	NM_022455.5(*NSD1*):c.3071C > G p.(Ser1024*)^f^	H	-	4	***NSD1***^**h**^				
					***ERCC2***	19-45868093-CACT--	NM_000400.4(*ERCC2*):c.594 + 2_594 + 5del p.?	rs762309206	4
6-43578333-C-T	NM_006502.3(*POLH*):c.1117C > T p.(Gln373*)	S	rs121908564	4	***POLH***				
					***MPL***	1-43817975-G--	NM_005373.3(*MPL*):c.1653 + 1del p.?	rs755257605	4

*Abbreviations: ALL* acute lymphoblastic leukemia, *SMN* second malignant neoplasm, *Variant ID* variant identifier.

^a^Physical positions of the variants are according to the human genome assembly GRCh37 (hg19).

^b^Categorical entity of the SMN is given as: hematologic (H), astrocytoma (A) or other solid entities (S). The SMN entities of ALL patients affected by (likely) pathogenic variants were as follows: brain tumors (astrocytoma (*n* = 3)), hematologic neoplasms (myelodysplastic syndrome (MDS, *n* = 5), non-Hodgkin Lymphoma (NHL, *n* = 2) and acute myeloid leukemia (AML, *n* = 1)) and other solid tumors (thyroid cancer (*n* = 2), mucoepidermoid carcinoma (*n* = 1), melanoma (*n* = 1) and nerve sheath tumor (*n* = 1)); for detailed clinical and variant information see Supplementary Tables 3, 4.

^c^Explains the variant pathogenicity and is given by commonly used scores: “likely pathogenic” classification is referred to as class 4 and “pathogenic” as class 5.

^d^Variant pathogenicity was assessed according to standard interpretation guidelines (see Supplementary Tables 2, 4 for details).

^e^One SMN patient who developed an astrocytoma carried two deleterious variants.

^f^These 5 patients deceased within 10 years after the SMN.

^g^These two variants were observed homozygous.

^h^Cancer predisposition related to these genes may follow an autosomal dominant inheritance mode, implicating that even heterozygous germline variants may be causative, whereas heterozygous variants in the other genes (only recessively linked to cancer risk) imply only a carrier status for related conditions (see Supplementary Table 3 for details).

^i^Patient was previously diagnosed with Neurofibromatosis type 1.

^j^Variant was also detected in a DNA-sample from a subsequently developed second SMN (CMML), but not in hair follicle derived DNA of the affected patient, indicating the involvement of clonal hematopoiesis.

Among the SMN patients, (likely) pathogenic variants in one of the candidate genes were observed in 3 of 11 (27%) cases with brain tumors, 5 of 28 (18%) with other solid tumors, and 8 of 36 (22%) with hematologic SMN. Three individuals with hematologic SMN carried variants in Ras signaling pathway genes (*LZTR1* and *KRAS*); for clinical details see Supplementary Table 3. One of these patients, determined with *LZTR1* variant rs869320686 p.(Gly248Arg), developed a myelodysplastic syndrome (MDS) 2.1 years after undergoing hyperdiploid pre B-cell ALL. Revision of the clinical reports revealed that this patient was diagnosed with mild developmental delay, facial anomalies, and a short stature. Suspected clonal hematopoiesis was confirmed for *KRAS* variant rs121913530, by analyzing hair follicle-derived DNA. Two male patients, identified with astrocytoma at 9.2 and 9.7 years after diagnosis of ALL, harbored deleterious variants in *ATM*. Two SMN patients carried variants in mismatch repair genes: one patient with a mucoepidermoid carcinoma (*MSH6*) and one individual with an astrocytoma (*PMS2*); the later also carried an *ATM* variant. One male patient with a melanoma 16 years after his pre B-cell ALL carried a variant in *A2ML1*, rs773034576. While conducting this study, the clinical significance of *A2ML1* variants related to the Noonan syndrome was refuted [8]. However, for this patient, this likely pathogenic variant was the only one we determined. The *TP53* variant, rs121912651, was observed in a female patient developing MDS at 8.8 years after diagnosis of ALL. Two observations, Pro470Valfs*3 (*PMS2*) and rs587776650 (*NBN*) were homozygous. All other variants were detected with a variant allele fraction of approximately 50% (i.e., heterozygous); for further details see Supplementary Tables 3 and 4.

Excluding the variants *KRAS* rs121913530 and *A2ML1* rs773034576, (likely) pathogenic variants in one of the CPG were 1.97-times more frequent in SMN patients (14/148 vs. 14/75 affected patients; *P*(*Pearson’s X*^2^) = 0.050). Conditional Cox-Regression analysis revealed that patients with a conspicuous finding in one of the candidate genes were at increased risk for SMN development (OR = 2.21, 95% CI = 0.989–4.944), *P* = 0.053 compared to the remaining patients.

Within the last decade, it became increasingly obvious that germline genetic variation plays an important role in the development of childhood cancers, including pediatric ALL. Moreover, cases of ALL plus subsequent SMN were reported in the context of familial cancer predisposition syndromes (CPS) such as the Li-Fraumeni syndrome or the *ETV6* deficiency [9, 10].

Previously published large-scale sequencing studies, including pediatric ALL patients, determined 4–5% of the patients with variants in CPG [11] or an underlying CPS [12]. In line, here we identified 14 (9%) of the ALL patients without SMN with unfavorable CPG variants, and in 6 of these cases (4% of the control patients) an underlying CPS may be suspected, either because the variant was determined to be homozygous (i.e. *NBN)* or due to an autosomal dominant inheritance mode of the associated phenotype (see Table 1 for details). Notably, this was also the case in 9 (12%) of the SMN patients in our study. It is important to note that patients with a monoallelic/heterozygous variant in a gene that is associated with an autosomal recessive disease only have a carrier status for this condition. Furthermore, the aim of this study was not to diagnose previously unrecognized CPS, but to describe the mutational landscape of variants in known CPGs and to identify potential indicators of increased SMN risk in pediatric ALL patients. Nevertheless, in addition to a specific variation, we identified some patients with additional characteristics consistent with features of suspected CPS: In particular, the male patient carrying the *LZTR1* variant rs869320686 p.(Gly248Arg), previously described to be associated with the Noonan syndrome [13], presented several additional features [14]. In contrast, other variants we observed were previously reported in the context of SMN development, e.g., the mutational hotspot *TP53* variant, rs121912651, which was previously observed in B-cell ALL patients with SMN [9]. Using remission bone marrow samples, we cannot completely exclude clonal hematopoiesis here. Hence, besides considering detailed individual clinical and familial information, an adequate diagnosis of CPS would require additional analyses to confirm our findings, e.g., sequencing of DNA derived from additional tissues. In the AIEOP-BFM ALL 2000 study, CPS screening was not part of the routine diagnostic. As we were not able to re-contact families for research purposes, incomplete information (e.g., scarce non-cancer phenotype data) is a clear limitation of our study. To our knowledge, only one of the patients included here was diagnosed with a CPS prior to the current candidate gene study (see Table 1). Therefore, our results, together with previous studies [12, 15], demonstrate that systematic CPS gene screening in pediatric ALL reveals higher proportions of predisposing germline variants in ALL and SMN patients than previously anticipated. In our study population, SMN occurred up to 16 years after diagnosis of ALL, and only 46 of 75 (61%) SMN patients were alive within 10 years of the SMN diagnosis (compare Fig. 1A, B and Supplementary Table 3). Moreover, since variation in certain CPG confers a moderately to substantially increased lifetime risk for cancer development (e.g., *CHEK2* vs. *TP53*), we cannot exclude that also patients in our control group may develop SMN in the future. Consistent with previous investigations [3], this underscores the importance of long-term follow-up and the development of novel treatment approaches – including specific preventive measures—through a better understanding of the underlying pathobiology in this specific patient group.

In conclusion, SMN patients in our study had a 2.0-fold higher frequency of (likely) pathogenic germline CPG variants, and the presence of such variants conferred a 2.2-fold increased risk of SMN in pediatric ALL patients. Nevertheless, the majority of SMN patients (>70%) had no deleterious alterations in any of the included candidate genes. In future studies, besides non-targeted exome-wide analyses, novel analytical approaches employing more collective use of genetic information may further enhance our understanding and improve the future profiling of SMN risk in ALL patients.

### Supplementary information

Supplementary Material

## Data Availability

All relevant information and data generated or analyzed during this study are included in this published article and its supplementary information files.
