# Supplementary material for: Germline variants in patients developing second malignant neoplasms after therapy for pediatric acute lymphoblastic leukemia—a case-control study
Source: Leukemia. 2024 Feb 27;38(4):887–92. doi: 10.1038/s41375-024-02173-2 (PMC10997515; doi:10.1038/s41375-024-02173-2)
Supplement: Supplementary file 1 — Supplementary Material [file 41375_2024_2173_MOESM1_ESM.pdf]

Supplementary Information to

Germline variants in patients developing second malignant neoplasms after therapy for pediatric acute lymphoblastic leukemia – a case-control study

Stefanie V. Junk[1, 2, §], Alisa Förster[3], Gunnar Schmidt[3], Martin Zimmermann[1], Birthe Fedders[4], Bernd Haermeyer[3], Anke K. Bergmann[3], Anja Möricke[4], Gunnar Cario[4], Bernd Auber[3], Martin Schrappe[4], Christian P. Kratz[1] and Martin Stanulla[1, §]

[1] Pediatric Hematology and Oncology, Hannover Medical School, Hannover, Germany
[2] Department of Pediatric Oncology, Hematology and Clinical Immunology, Medical Faculty, Heinrich Heine University Düsseldorf, Düsseldorf, Germany
[3] Institute of Human Genetics, Hannover Medical School, Hannover, Germany
[4] Department of Pediatrics, University Hospital Schleswig-Holstein, Kiel, Germany
[§] corresponding authors

Supplementary Appendix Content

Supplementary Methods

Supplementary Tables

Suppl. Table 1. Characteristics of all 223 ALL patients included in this candidate gene approach, according to their SMN status.

Suppl. Table 2. Selected candidate genes.

Suppl. Table 3. Detailed clinical information on patients in our study population with (likely) pathogenic variants in the candidate genes.

Suppl. Table 4: Details on determined (likely) pathogenic variants and their pathogenicity classification.

Supplementary References

Supplementary Figures

Suppl. Figure 1: Distribution of classified variants by case-control status of the carrier.

Supplementary Methods

Candidate gene sequencing

We performed whole exome sequencing (WES) on a NovaSeq sequencing platform (Illumina GmbH, Berlin, Germany) using an IDT xGen Exome Research Panel V2 (Integrated DNA Technologies, Inc., Leuven, Belgium); annotation and final filtering on the 159 candidate genes was carried out with VarSeq 2.3 (Golden Helix, Inc., Bozeman, USA). In sequencing analyses of the exons (±70 base pairs), minimum depth was 100x. Copy number variations were not analyzed in this WES-based approach. The classification of pathogenicity according to the ACMG/AMP criteria and additional refinements of the variants was performed independently by two investigators and then extensively discussed in the context of the available literature and database information (gnomAD, ClinVar, ClinGen, LOVD3, INSiGHT, BRCA-XChange, etc.). Whenever available curated classifications based on the reviews of ClinGen expert panels were included. The classifications presented here are the result of this evaluation process. Overall, a total of 643 variants were determined and classified: 248(38.57%) in the finally included 75 cases and 395(61.43%) in the 148 control patients. According to Pearson's Chi-squared testing, the observed variant frequencies by pathogenicity tiers were not significantly different from the expected; further details can be obtained from Suppl. Figure 1.

Statistical analyses

Odds ratio (OR) was calculated by performing conditional Cox-Regression analysis with SPSS ("COXREG" procedure), an equivalent to conditional logistic regression analysis[1]. This procedure accounts for the matching aspect (case to control ratio 1:2) of our study design; we successfully validated our result with SAS ("SAS proc phreg" procedure).

Supplementary Table 1. Characteristics of all 223 ALL patients included in this candidate gene approach, according to their SMN status.

		Patients with ALL (n=148), (n%)	Patients with ALL and SMN (n=75), (n%)	$P(X^2)$[a]
Sex	Male	95(64%)	45(60%)	
	Female	53(36%)	30(40%)	0.541
Age at diagnosis of ALL [y]	< 6	76(51%)	38(51%)	
	≥ 6 <10	23(16%)	14(19%)	
	≥ 10	49(33%)	23(31%)	0.823
Immunophenotype	B-cell ALL	105(71%)	51(68%)	
	T-cell ALL	43(29%)	23(31%)	0.755
	Other/not characterized[b]	0(0%)	1(1%)	
Initial white blood cell count [/µL]	< 10000	53(36%)	23(31%)	
	≥ 10000 < 50000	49(33%)	21(28%)	
	≥ 50000 < 100000	19(13%)	6(8%)	
	≥ 100000	23(16%)	13(17%)	0.791
	Unknown	4(3%)	12(16%)	
Hyperdiploidy[c]	Yes	18(12%)	9(12%)	
	No	103(70%)	50(67%)	0.947
	Unknown	27(18%)	16(21%)	
CNS positivity[d]	Yes	8(5%)	3(4%)	
	No	119(80%)	49(65%)	0.893
	Unknown	21(14%)	23(31%)	
Cranial irradiation[e]	Yes	70(47%)	36(48%)	
	No	78(53%)	39(52%)	0.921
MRD risk group[f]	Standard	44(30%)	24(32%)	
	Intermediate	68(46%)	33(44%)	
	High	25(17%)	13(17%)	0.938
	Unknown	11(7%)	5(7%)	
Final risk group[g]	Standard risk	28(19%)	15(20%)	
	Intermediate risk	63(43%)	32(43%)	0.976
	High risk	57(39%)	28(37%)	
Patients with a conspicuous finding[h]	Yes	14(9%)	15(20%)	
	No	134(91%)	60(80%)	0.027

Abbreviations: Acute lymphoblastic leukemia (ALL), second malignant neoplasm (SMN), central nervous system (CNS), minimal residual disease (MRD).

[a] *P*-values resulting from X^2 or Fisher's exact test: Patients of our study population with ALL and subsequent SMN versus ALL patients without SMN.

[b] The immunophenotype of one patient was not available to us.

[c] Defined by cytogenetics (> 50 chromosomes) or by flow cytometric analyses of the ratio of DNA content of leukemic G0/G1 cells to normal diploid lymphocytes (≥ 1.16).

[d] CNS negative, puncture nontraumatic without leukemic blasts in the cerebrospinal fluid (CSF) after cytocentrifugation; CNS positive, puncture nontraumatic with >5 leukocytes /µL in the CSF with identifiable blasts.

[e] Preventive cranial irradiation (CI) at 12 Gy was only applied in T-cell ALL and high-risk patients; CNS-positive patients received 18 Gy (<2 years, 12 Gy; <1 year, no CI).

[f] Risk stratification based on MRD analysis for ERG: Standard risk, MRD-negative on treatment day33 and 78; high risk, leukemic cell load $5x10^{-4}$ on treatment day 78; all other results correspond to intermediate risk.

[g] Treatment group according to risk stratification including all relevant diagnostic parameters.

[h] A (likely) pathogenic germline variant in one of the candidate genes was considered as a conspicuous finding; one of the SMN patients was determined with findings in two distinct genes, and one pathogenic *KRAS* (rs121913530) variant, determined in another SMN patient was identified to be of somatic origin and therefore not counted (compare Table 1 and Suppl. Table 3).

Supplementary Table 2. Selected candidate genes.

Gene[a]	Panel[b]	Gene ID[c]	Associated phenotype	Inheritance[d]
ABL1[2]	§	25	Leukemia associated (Philadelphia chromosome), Familial congenital heart defects and skeletal malformations	AD
AFF3[2]	§	3899	*MLL*/*AF4* rearrangement infant ALL: poor prognosis, Top associated region (preliminary data, genome wide association study (GWAS) ALL vs. SMN)	AR
AKT1[2]	§	207	Cowden syndrome 6, T-cell ALL	AR
ARID5B[2]	§	84159	Increased genetic susceptibility to ALL	AD / AR
CDKN2B[2]	§	1030	ALL-associated SNV, SNV of prognostic significance for acute promyelocytic leukemia	AD
CHEK2[3]	§	11200	hereditary breast and ovarian cancer, hereditary SNV are associated with hematologic neoplasms (association to Li-Fraumeni syndrome is outdated)[4]	AD
FLT3[2]	§	2322	Acute myeloid leukemia (somatic variants)	AD
GATA3[2]	§	2625	Increased genetic susceptibility to ALL	AD
IKZF3[2]	§	22806	Somatic mutations, near haploid and low hypodiploid ALL, CML, Immunodeficiency	AD
JAK1[2]	§	3716	Increased susceptibility to ALL, *BCR-ABL1*-negative, HR patients with SNV: poor prognosis.	AD
JAK2[2]	§	3717	ALL-associated SNV, *BCR-ABL1*-negative, HR patients: poor prognosis, thrombocytemia	AR
JAK3[2]	§	3718	Increased susceptibility to ALL, *BCR-ABL1*-negative, HR patients with SNV: poor prognosis.	AR
NOTCH1[2]	§	4851	T-cell ALL-associated somatic mutations, prognosis relevant	AR
PDGFRB[2]	§	5159	Familial infantile myofibromatosis and brain calcification, chromosomal rearrangements in ALL and AML	AD
ALK[2]	§, ɫ	238	Neuroblastoma, susceptibility to, 3	AD
APC[2]	§, ɫ	324	Familial Adenomatous Polyposis	AD
CDH1[5]	§, ɫ	999	Familial diffuse gastric cancer with or without cleft lip and/or palate	AD
CDKN2A[2]	§, ɫ	1029	Familial atypical multiple mole melanoma-pancreatic carcinoma syndrome (FAMMPC)	AD
ETV6[2]	§, ɫ	2120	Thrombocytopenia 5	AD
IKZF1[2]	§, ɫ	10320	Common variable immunodeficiency 13	AD
NF1[2]	§, ɫ	4763	Neurofibromatosis type 1	AD
NF2[2]	§, ɫ	4771	Neurofibromatosis type 2	AD
PAX5[2]	§, ɫ	5079	Susceptibility to acute lymphoblastic leukemia	AD
PTEN[6]	§, ɫ	5728	Cowden syndrome 1	AD
RB1[2]	§, ɫ	5925	Retinoblastoma	AD
RET[2]	§, ɫ	5979	Multiple endocrine neoplasia type 1A and 2B (MEN2A, MEN2B)	AD
RUNX1[7]	§, ɫ	861	Acute myeloid leukemia	AD
TP53[8]	§, ɫ	7157	Li-Fraumeni syndrome	AD / AR
BRAF[2]	§, ɫ	673	Noonan (like) syndrome / rasopathy	AD
BRCA1[3]	§, ɫ	672	Breast and ovarian cancer	AR
BRCA2[3]	§, ɫ	675	familial breast and ovarian cancer, medulloblastoma, glioblastoma, Wilms tumor (AD)/ Fanconi anemia(AR)	AD / AR
FANCA[2]	§, ɫ	2175	Fanconi anemia	AR
FANCB[2]	§, ɫ	2187	Fanconi anemia	AD
KRAS[2]	§, ɫ	3845	Noonan (like) syndrome / rasopathy	AD
MSH2[9]	§, ɫ	4436	Lynch / Constitutional mismatch repair deficiency syndrome	AD / AR
MSH6[9]	§, ɫ	2956	Lynch / Constitutional mismatch repair deficiency syndrome	AD / AR
NRAS[2]	§, ɫ	4893	Noonan (like) syndrome / rasopathy	AD
PALB2[3]	§, ɫ	79728	Fanconi anemia	AD
PMS2[9]	§, ɫ	5395	Lynch / Constitutional mismatch repair deficiency syndrome	(AD)/AR
PTCH1[2]	§, ɫ	5727	Gorlin syndrome	AD
SDHA[2]	§, ɫ	6389	Paraganglioma / pheochromocytoma (genes operating within pathway/cluster 1)	AD
SDHB[2]	§, ɫ	6390	Paraganglioma / pheochromocytoma (genes operating within pathway/cluster 1)	AD / AR

Gene[a]	Panel[b]	Gene ID[c]	Associated phenotype	Inheritance[d]
VHL[2]	§, ǂ	7428	Paraganglioma / pheochromocytoma (genes operating within pathway/cluster 1)	AD / AR
ABCB11[2]	ǂ	8647	Familial intrahepatic cholestasis	AD
AIP[2]	ǂ	9049	Pituitary adenoma predisposition	AR
ATM[3]	ǂ	472	*Ataxia telangiectasia* / susceptibility to breast cancer	AD
BAP1[2]	ǂ	8314	*BAP1* tumor predisposition syndrome	AD / AR
BLM[2]	ǂ	641	Bloom syndrome	AD
CD27[2]	ǂ	939	Lymphoproliferative syndrome 2	AR
CD70(TNSF7)[2]	ǂ	970	Lymphoproliferative syndrome 3	AR
CDC73[2]	ǂ	79577	Hyperparathyroidism-jaw tumor syndrome	AR
CDKN1C[2]	ǂ	1028	Beckwith-Wiedemann syndrome; IMAGE syndrome	AD
CEBPA[2]	ǂ	1050	Acute myeloid leukemia	AD
CREBBP[2]	ǂ	1387	Rubinstein-Taybi syndrome	AD
CTLA4[2]	ǂ	1493	Autoimmune lymphoproliferative syndrome, type V	AD
CTR9[2]	ǂ	9646	Wilms tumor predisposition	AD
DICER[2]*1*	ǂ	23405	*DICER1* tumor predisposition syndrome	AD
DIS3L2[2]	ǂ	129563	Perlman syndrome	AD
EZH2[2]	ǂ	2146	Weaver syndrome	AR
FAS[2]	ǂ	355	Autoimmune lymphoproliferative syndrome, type IA	AD
FBXW7[2]	ǂ	55294	Wilms tumor predisposition	AD
GATA2[2]	ǂ	2624	Emberger syndrome / immunodeficiency 21 / AML, susceptibility to	AD
GPC3[2]	ǂ	2719	Simpson-Golabi-Behmel syndrome 1	AD
GPR161[2]	ǂ	23432	Medulloblastoma	XLR
HAVCR2[2]	ǂ	84868	T-cell lymphoma, subcutaneous panniculitis like	AD
HRAS[2]	ǂ	3265	Costello syndrome, epidermal nevus, bladder cancer, head and neck squamous cell carcinoma	AR
IKBKAP(ELP1)[2]	ǂ	8518	Medulloblastoma	AD / *de novo*
ITK[2]	ǂ	3702	*ITK* deficiency syndrome	AD / *de novo*
LIG4[2]	ǂ	3981	*LIG4* syndrome	AR
MEN1[2]	ǂ	4221	Multiple endocrine neoplasia 1	AR
NBN[2]	ǂ	4683	Nijmegen Breakage syndrome	AD
NSD1[2]	ǂ	64324	Sotos syndrome	AR
PHOX2B[2]	ǂ	8929	Central hypoventilation syndrome, neuroblastoma	AD
PIK3CA[2]	ǂ	5290	Cowden syndrome 5, wide range of somatic mutations in cancer	AD
RECQL4[2]	ǂ	9401	Baller-Gerold syndrome / RAPADILINO syndrome / Rothmund-Thomson syndrome	AD
REST[2]	ǂ	5978	Wilms tumor	AR
RMRP[2]	ǂ	6023	Cartilage-hair hypoplasia	AD
SAMD9[2]	ǂ	54809	Mirage syndrome (AD); tumoral calcinosis (AR)	AD / AR
SAMD9L[2]	ǂ	219285	Ataxia-pancytopenia syndrome	AD / AR
SETBP1[2]	ǂ	26040	Schinzel-Giedion syndrome	AD / AR
SH2D1A[2]	ǂ	4068	Lymphoproliferative syndrome, X-linked, 1	AD / AR
SMARCA4[2]	ǂ	6597	Rhabdoid tumor predisposition syndrome 2 / Coffin Siris syndrome 4	AD / AR
SMARCB1[2]	ǂ	6598	Rhabdoid tumor predisposition syndrome 1 / Coffin Siris syndrome / schwannomatosis	AR
SMARCE1[2]	ǂ	6605	Coffin Siris syndrome 5 / meningioma (susceptibility to)	AR
STK11[2]	ǂ	6794	Peutz-Jeghers syndrome	AR
TRIM28[2]	ǂ	10155	Wilms tumor predisposition	AR
TRIM37[2]	ǂ	4591	Mulibrey nanism	AD
TSC1[2]	ǂ	7248	Tuberous sclerosis I	AD

Gene[a]	Panel[b]	Gene ID[c]	Associated phenotype	Inheritance[d]
TSC2[2]	ǂ	7249	Tuberous sclerosis II	AD
USB1[2]	ǂ	79650	Poikiloderma with neutropenia	AD
WAS[2]	ǂ	7454	Wiskott-Aldrich syndrome	AD
WT1[2]	ǂ	7490	Wilms tumor 1	AD
A2ML1[2]	ǂ	144568	Noonan (like) syndrome / rasopathy (outdated information)[10]	AR
ACD[2]	ǂ	65057	Dyskeratosis congenita	AR
BRIP1[3]	ǂ	83990	Fanconi anemia	AR
BUB1B[2]	ǂ	701	Mosaic variegated aneuploidy syndrome	AR
CBL[2]	ǂ	867	Noonan (like) syndrome / rasopathy	AR
CEP57[2]	ǂ	9702	Mosaic variegated aneuploidy syndrome	AR
CTC1[2]	ǂ	80169	Dyskeratosis congenita	AD
DDB2[2]	ǂ	1643	Xeroderma pigmentosum	AD
DKC1[2]	ǂ	1736	Dyskeratosis congenita	AD
EGLN1[2]	ǂ	54583	Paraganglioma / pheochromocytoma (genes operating within pathway/cluster 1)	AR
EGLN2[2]	ǂ	112398	Paraganglioma / pheochromocytoma (genes operating within pathway/cluster 1)	AR
EPAS1(HIF2A)[2]	ǂ	2034	Paraganglioma / pheochromocytoma (genes operating within pathway/cluster 1)	AR
EPCAM[2]	ǂ	4072	Lynch / Constitutional mismatch repair deficiency syndrome	AD
ERCC2[2]	ǂ	2068	Xeroderma pigmentosum	AD
ERCC3[2]	ǂ	2071	Xeroderma pigmentosum	AD
ERCC4[2]	ǂ	2072	Causing both Fanconi anemia and Xeroderma pigmentosum	AD
ERCC5[2]	ǂ	2073	Xeroderma pigmentosum	AD
FANCC[2]	ǂ	2176	Fanconi anemia	AD
FANCD2[2]	ǂ	2177	Fanconi anemia	AD
FANCE[2]	ǂ	2178	Fanconi anemia	AD
FANCF[2]	ǂ	2188	Fanconi anemia	AD
FANCG[2]	ǂ	2189	Fanconi anemia	AD
FANCI[2]	ǂ	55215	Fanconi anemia	AD
FANCL[2]	ǂ	55120	Fanconi anemia	AD
FH[2]	ǂ	2271	Paraganglioma / pheochromocytoma (genes operating within pathway/cluster 1)	AD
LZTR1[2]	ǂ	8216	Noonan (like) syndrome / rasopathy	AD
MAP2K1[2]	ǂ	5604	Noonan (like) syndrome / rasopathy	AR
MAP2K2[2]	ǂ	5605	Noonan (like) syndrome / rasopathy	AR
MLH1[9]	ǂ	4292	Lynch / Constitutional missmatch repair deficiency syndrome	AR
NHP2[2]	ǂ	55651	*Dyskeratosis congenita*	AD
NOP10[2]	ǂ	55505	*Dyskeratosis congenita*	AD
PARN[2]	ǂ	5073	*Dyskeratosis congenita*	AD
POLH[2]	ǂ	5429	*Xeroderma pigmentosum*	AD
PTCH2[2]	ǂ	8643	Gorlin syndrome	AD
PTPN11[2]	ǂ	5781	Noonan (like) syndrome / rasopathy	AD
RAD51C[3]	ǂ	5889	Fanconi anemia	AD
RAF1[2]	ǂ	5894	Noonan (like) syndrome / rasopathy, therapy-related AML	AD
RIT1[2]	ǂ	6016	Noonan (like) syndrome / rasopathy	AD
RPL11[2]	ǂ	6135	Diamond Blackfan anemia	AD
RPL35A[2]	ǂ	6165	Diamond Blackfan anemia	AR
RPL5[2]	ǂ	6083	Diamond Blackfan anemia	AR
RPS10[2]	ǂ	6204	Diamond Blackfan anemia	AR

Gene[a]	Panel[b]	Gene ID[c]	Associated phenotype	Inheritance[d]
RPS17[2]	ɫ	6218	Diamond Blackfan anemia	AR
RPS19[2]	ɫ	6223	Diamond Blackfan anemia	AR
RPS24[2]	ɫ	6229	Diamond Blackfan anemia	AR
RPS26[2]	ɫ	6231	Diamond Blackfan anemia	AD
RPS7[2]	ɫ	6201	Diamond Blackfan anemia	AR
RTEL1[2]	ɫ	51750	Dyskeratosis congenita	AR
SDHAF2[2]	ɫ	54949	Paraganglioma / pheochromocytoma (genes operating within pathway/cluster 1)	AD / AR
SDHC[2]	ɫ	6391	Paraganglioma / pheochromocytoma (genes operating within pathway/cluster 1)	AD / AR
SDHD[2]	ɫ	6392	Paraganglioma / pheochromocytoma (genes operating within pathway/cluster 1)	AD / AR
SHOC2[2]	ɫ	8036	Noonan (like) syndrome / rasopathy	AD / AR
SLX4[2]	ɫ	84464	Fanconi anemia	AD / AR
SOS1[2]	ɫ	6654	Noonan (like) syndrome / rasopathy	AR
SUFU[2]	ɫ	51684	Gorlin syndrome	AR
TERC[2]	ɫ	7012	Dyskeratosis congenita	AR
TERT[2]	ɫ	7015	Dyskeratosis congenita	AD
TINF2[2]	ɫ	26277	Dyskeratosis congenita	AD
TRIP13[2]	ɫ	9319	Mosaic variegated aneuploidy syndrome	AD
WRAP53[2]	ɫ	55135	Dyskeratosis congenita	AD
XPA[2]	ɫ	7507	Xeroderma pigmentosum	AR
XPC[2]	ɫ	7508	Xeroderma pigmentosum	AR
TPMT[2]	§	7172	Poor metabolism of thiopurines-1, cytotoxic side effects and poor outcome (ALL therapy)	AR
CALR[2]	§	811	Somatic mutations are associated with myeloproliferative neoplasms (MPN)[11-13]	AD / AR
MPL[2]	§	4352	Germline/somatic mutations are associated with myeloproliferative neoplasms (MPN)[11-13]	AD / AR

Abbreviations: Acute lymphoblastic leukemia (ALL), acute myeloid leukemia (AML), myelodysplastic syndrome (MDS), Non-B-cell non-Hodgkin lymphoma (NB-NHL), non-Hodgkin lymphoma (NHL), second malignant neoplasm (SMN), single nucleotide variants (SNV).

[a] Approved gene symbols, according to the human gene nomenclature committee (HGNC); all variants determined in this project were assessed according to standard variant interpretation guidelines as indicated above for each gene; variants in the mismatch repair genes were classified according to the most recent version of the “InSiGHT Variant Interpretation Committee MMR gene variant classification criteria” published in 2018 (see https://www.insight-group.org/criteria/).

[b] Included candidate genes were either considered to be relevant by us in previous investigations (§) or recommended by Byrjalsen *et al* 2021[14] (ɫ).

[c] Approved gene identifier (Entrez ID/GeneID; National Center for Biotechnology Information (NCBI)’s database for gene-specific information).

[d] Known inheritance modes: autosomal dominant (AD), autosomal recessive (AR) and X-linked recessive (XLR); patients with a heterozygous deleterious mutation in a gene related to conditions/CPS with a known AR inheritance mode were considered to have a carrier status.

Supplementary Table 3. Detailed clinical information on patients in our study population with (likely) pathogenic variants in the candidate genes.

Patient ID	Sex	Age at diagnosis of ALL	Final risk group[a]	Initial white blood cell count[/µL][b]	Immuno-phenotype	Hyper-diploidy[c]	Cranial irradiation[d]	SMN entity	Time to SMN[e]	Survival status	Finding and molecular consequence	Depth[f]	VAF
230	Male	1.6	SR	< 50000	B	Yes	No	AML	2.3	deceased	NM_007194.4(*CHEK2*):c.349A>G p.(Arg117Gly)[g]	493	0.525
35	Male	13.1	IR	Unknown	B	No	No	MDS	2.9	alive	NM_000059.4(*BRCA2*):c.1813dup p.(Ile605Asnfs*11)	364	0.462
175	Male	2.1	IR	< 50000	B	Yes	No	MDS	2.1	deceased	NM_006767.4(*LZTR1*):c.742G>A p.(Gly248Arg)	329	0.514
205	Male	10.2	IR	< 50000	B	No	No	MDS	2.5	alive	NM_006767.4(*LZTR1*):c.401-2_401-1del p.?	139	0.525
265	Male	7.6	IR	Unknown	B	Unknown	No	MDS	9.9	alive	NM_016038.4(*SBDS*):c.184A>T p.(Lys62*)	55	0.345
350	Female	12.8	IR	< 50000	B	Unknown	No	MDS	8.8	deceased	NM_000546.6(*TP53*):c.742C>T p.(Arg248Trp)[h]	248	0.504
150	Male	8.3	HR	≥ 50000	T	No	No	NB-NHL	7.5	deceased	NM_022455.5(*NSD1*):c.3071C>G p.(Ser1024*)	341	0.507
220	Female	11.3	HR	Unknown	B	No	Yes	NHL	4.7	alive	NM_004985.5(*KRAS*):c.34G>C p.(Gly12Arg)[i]	237	0.451
80	Male	3.0	IR	≥ 50000	B	No	No	Astrocytoma	5.1	alive	NM_000267.3(*NF1*):c.1246C>T p.(Arg416*)	145	0.393
90	Male	2.2	SR	< 50000	B	No	No	Astrocytoma	9.7	alive	NM_000535.7(*PMS2*):c.1408delins47 p.(Pro470Valfs*3)	176	0.813
											NM_000051.4(*ATM*):c.6199-1G>T p.?	91	0.615
160	Male	2.6	IR	≥ 50000	B	No	No	Astrocytoma	9.1	deceased	NM_000051.4(*ATM*):c.802C>T p.(Gln268*)	161	0.472
60	Male	15.8	HR	≥ 50000	T	Unknown	Yes	Nerve sheath tumor	10.6	alive	NM_016038.4(*SBDS*):c.258+2T>C p.?	89	0.494
105	Male	4.1	SR	< 50000	B	No	No	Mucoepidermoid carcinoma	9.4	alive	NM_000179.3(*MSH6*):c.3261dup p.(Phe1088Leufs*5)	76	0.434
240	Male	1.4	HR	≥ 50000	B	Unknown	Yes	Melanoma	15.9	alive	NM_144670.6(*A2ML1*):c.2764+1G>A p.?	94	0.351
45	Male	2.7	HR	< 50000	B	Yes	Yes	Thyroid cancer	11	alive	NM_004380.3(*CREBBP*):c.1447C>T p.(Arg483*)	288	0.396
315	Female	4.0	SR	< 50000	B	No	Yes	Thyroid cancer	6.3	alive	NM_006502.3(*POLH*):c.1117C>T p.(Gln373*)	182	0.500
32	Male	13.5	IR	< 50000	B	No	No	None	-	alive	NM_002834.5(*PTPN11*):c.173A>G p.(Asn58Ser)	127	0.622
59	Male	16.0	HR	≥ 50000	T	No	Yes	None	-	alive	NM_000400.4(*ERCC2*):c.594+2_594+5del p.?	196	0.495
86	Male	2.7	SR	< 50000	B	Yes	No	None	-	alive	NM_016038.4(*SBDS*):c.258+2T>C p.?	149	0.463
134	Female	3.1	SR	≥ 50000	B	No	No	None	-	alive	NM_000267.3(*NF1*):c.1246C>T p.(Arg416*)	183	0.437
161	Male	7.3	HR	Unknown	T	No	Yes	None	-	alive	NM_033084.6(*FANCD2*):c.3481C>T p.(Gln1161*)	150	0.487

Patient ID	Sex	Age at diagnosis of ALL	Final risk group[a]	Initial white blood cell count[/µL][b]	Immuno-phenotype	Hyper-diploidy[c]	Cranial irradiation[d]	SMN entity	Time to SMN[e]	Survival status	Finding and molecular consequence	Depth[f]	VAF
206	Female	2.2	HR	< 50000	T	No	Yes	None	-	alive	NM_007294.4(*BRCA1*):c.5503C>T p.(Arg1835*)	229	0.480
251	Male	14.7	IR	< 50000	B	No	No	None	-	alive	NM_005373.3(*MPL*):c.1653+1del p.?	175	0.423
259	Male	6.8	IR	< 50000	T	Unknown	No	None	-	alive	NM_004260.4(*RECQL4*):c.2464-1G>C p.?	267	0.476
273	Male	11.0	IR	≥ 50000	T	No	Yes	None	-	alive	NM_004360.5(*CDH1*):c.2200A>T p.(Arg734*)	230	0.530
293	Male	9.4	HR	< 50000	B	No	Yes	None	-	alive	NM_006767.4(*LZTR1*):c.2062C>T p.(Arg688Cys)	220	0.514
307	Female	3.6	HR	< 50000	B	Unknown	Yes	None	-	alive	NM_007194.4(*CHEK2*):c.349A>G p.(Arg117Gly)[e]	258	0.500
328	Male	5.0	HR	< 50000	T	No	Yes	None	-	alive	NM_002485.5(*NBN*):c.657_661del p.?	35	1.00
333	Female	12.4	HR	≥ 50000	B	No	Yes	None	-	alive	NM_016038.4(*SBDS*):c.258+2T>C p.?	124	0.452
377	Female	11.7	IR	< 50000	B	No	No	None	-	alive	NM_033084.6(FANCD2):c.3289C>T p.(Arg1097*)	114	0.404

Abbreviations: Acute lymphoblastic leukemia (ALL), second malignant neoplasm (SMN), single nucleotide variants (SNV), central nervous system (CNS), identifier (ID), minor allele frequency (MAF), variant allele fraction (VAF).

[a] Treatment group according to risk stratification including all relevant diagnostic parameters; groups were high (HR), intermediate (IR) and standard risk (SR).

[b] White blood cell count at diagnosis of ALL.

[c] Defined by cytogenetics (> 50 chromosomes) or by flow cytometric analyses of the ratio of DNA content of leukemic G0/G1 cells to normal diploid lymphocytes (≥ 1.16).

[d] Preventive cranial irradiation (CI) at 12 Gy was only applied in T-cell ALL and high-risk patients; CNS-positive patients received 18 Gy (<2 years, 12 Gy; <1 year, no CI).

[e] Time from diagnosis of ALL to diagnosis of SMN [years].

[f] Highest MAF observed in a subpopulation of gnomAD (v.2.1.1, exomes): non-Finnish European (NFE), African/African American (AFR), South Asian (SAS).

[g] This rare *CHEK2* variant, rs28909982 (c.349A>G p.(Arg117Gly), present in patients from both groups was previously identified to moderately increase the risk of hereditary breast cancer but not of ovarian cancer[15,16].

[h] This *TP53* variant, rs121912651 (c.742C>T p.(Arg248Trp) is a frequent germline variant related to the Li-Fraumeni syndrome[17]. It was determined here with a VAF of 50% (125/248) and not considered to be caused by clonal hematopoiesis; DNA samples from additional tissues for subsequent testing were not available to us, due to the patients' death.

[i] This *KRAS* variant, rs121913530 (c.34G>C p.(Gly12Arg, VAF = 45%), was considered to be of somatic origin and therefore excluded from subsequent frequency calculations. As germline variants at this residue are rare and the patient showed no Noonan (like) syndrome related conditions, additional DNA from hair follicles was analyzed to test for clonal hematopoiesis. Nevertheless, *KRAS* codon 12 is known to be frequently mutated in human cancers and associated with a poor prognosis in certain entities[18].

Supplementary Table 4: Details on determined (likely) pathogenic variants and their pathogenicity classification.

Patient ID	Finding and molecular consequence	Highest MAF[a]	REVEL[b]	CADD[b]	SpliceAI[b]	BayesDel (no AF)[b]	ACMG/AMP criteria met[c,d]	Classification according to the applicable refinement	Variant reviewed / curated by expert panel[e]	Final classification[d]
230	NM_007194.4(*CHEK2*):c.349A>G p.(Arg117Gly)	0.000203 (NFE)	0.930	26.0	0.00	0.54	(not applicable)	class 4 (resides in functional domain and is considered to be clinically relevant)[3]	https://databases.lovd.nl/shared/variants/0000148824#00024043; ClinVar: 37 submissions:9x P, 28x LP	LP
35	NM_000059.4(*BRCA2*):c.1813dup p.(Ile605Asnfs*11)	0.000066 (AFR)	-	-	0.00	-	(not applicable)	class 5 (loss of function variant)[3]	https://brcaexchange.org/variant/908057	P
175	NM_006767.4(*LZTR1*):c.742G>A p.(Gly248Arg)	(absent)	0.839	28.9	0.00	0.29	PVS1, PS1, PP3, PM2	-	(no expert panel review available); ClinVar: 9 submissions: 8x P	P
205	NM_006767.4(*LZTR1*):c.401-2_401-1del p.?	0.000055 (SAS)	-	33.0	1.00	-	PVS1, PM2	-	(no expert panel review available); ClinVar: 2 submissions: 2x LP	LP
265	NM_016038.4(*SBDS*):c.184A>T p.(Lys62*)	0.000326 (SAS)	-	44.0	0.26	0.66	PVS1, PS3, PM3	-	(no expert panel review available); ClinVar: 6 submissions: 6x P	P
350	NM_000546.6(*TP53*):c.742C>T p.(Arg248Trp)	0.000009 (NFE)	0.927	27.0	0.00	0.54	(PS3, PS4_supporting, PM1, PP3_moderate, PM2_supporting)	ACMG/AMP criteria can be applied, as specified[8]	https://erepo.clinicalgenome.org/evrepo/ui/classification/291eceb4-ecb0-489a-b637-b9ff4f5f34d5	P
150	NM_022455.5(*NSD1*):c.3071C>G p.(Ser1024*)	(absent)	-	-	0.00	0.66	PVS1, PM2	-	(no expert panel review available, not in ClinVar)	LP
220	NM_004985.5(*KRAS*):c.34G>C p.(Gly12Arg)	(absent)	0.821	29.6	0.00	0.30	PS3, PM1, PM2, PM5, PP2, PP3	-	(no expert panel review available); ClinVar: 2 submissions: 2x P	P
80	NM_000267.3(*NF1*):c.1246C>T p.(Arg416*)	0.000009 (NFE)	-	35.0	0.03	0.60	PVS1, PS4, PM2	-	(no expert panel review available); ClinVar: 16 submissions: 16x P	P
90	NM_000535.7(*PMS2*):c.1408delins47 p.(Pro470Valfs*3)	(absent)	-	-	-	-	(not applicable)	class 5 (stop gain before last exon)[9]	(no expert panel review available, not in ClinVar)	P
	NM_000051.4(*ATM*):c.6199-1G>T p.?	(absent)	-	35.0	0.98	0.40	(not applicable)	class 4 (impairing splice site variant, C-Terminus FATKIN domain)[3]	https://clinicalgenome.org/site/assets/files/7392/clingen_hbop_acmg_specifications_atm_v1-1.pdf	LP
160	NM_000051.4(*ATM*):c.802C>T p.(Gln268*)	0.000009 (NFE)	-	36.0	0.03	0.62	(not applicable)	class 5 (truncating variant before FATKIN domain)[3]	(no expert panel review available); ClinVar: 3 submissions: 2x P, 1x LP	P
60	NM_016038.4(*SBDS*):c.258+2T>C p.?	0.009576 (NFE)	-	35.0	0.93	0.66	PVS1, PS3, PS4	-	(no expert panel review available); ClinVar: 50 submissions: 45x P, 3x LP, 1x risk factor, 1x not provided	P
105	NM_000179.3(*MSH6*):c.3261dup p.(Phe1088Leufs*5)	0.000124 (AFR)	-	24.9	0.00	-	(not applicable)	class 5 (variation results in a stop gain)[9]	http://www.insight-database.org/classifications/index.html?gene=MSH6&variant=&protein=Phe1088Leufs*5	P
240	NM_144670.6(*A2ML1*):c.2764+1G>A p.?	(absent)	-	33.0	0.86	0.21	PVS1, PM2	-	(no expert panel review available, not in ClinVar)	LP
45	NM_004380.3(*CREBBP*):c.1447C>T p.(Arg483*)	(absent)	-	37.0	0.00	0.66	PVS1, PS4, PM2	-	(no expert panel review available); ClinVar: 1 submission: 1x P	P
315	NM_006502.3(*POLH*):c.1117C>T p.(Gln373*)	0.000004 (NFE)	-	36.0	0.00	0.66	PVS1, PM2	-	(no expert panel review available); ClinVar: 1 submission: 1x P	LP
32	NM_002834.5(*PTPN11*):c.173A>G p.(Asn58Ser)	0.000079 (NFE)	0.610	23.5	0.00	-0.18	PM1, PM2, PM5, PP2	-	(no expert panel review available); ClinVar: 1 submission: 1x LP	LP
59	NM_000400.4(*ERCC2*):c.594+2_594+5del p.?	0.000123 (NFE)	-	-	0.94	-	PVS1, PS3, PM3	-	(no expert panel review available); ClinVar: 4 submissions: 1x P, 3x LP	LP
86	NM_016038.4(*SBDS*):c.258+2T>C p.?	0.009576 (NFE)	-	35.0	0.93	0.66	PVS1, PS3, PS4	-	(no expert panel review available); ClinVar: 50 submissions: 45x P, 3x LP, 1x risk factor, 1x not provided	P

Patient ID	Finding and molecular consequence	Highest MAF[a]	REVEL[b]	CADD[b]	SpliceAI[b]	BayesDel (no AF)[b]	ACMG/AMP criteria met[c,d]	Classification according to the applicable refinement	Variant classification reviewed/ curated by expert panel[d]	Final classifi-cation[d]
134	NM_000267.3(*NF1*):c.1246C>T p.(Arg416*)	0.000009 (NFE)	-	35.0	0.03	0.60	PVS1, PM2	-	(no expert panel review available), ClinVar: 16 submissions: 16x P	P
161	NM_033084.6(*FANCD2*):c.3481C>T p.(Gln1161*)	0.000026 (NFE)	-	43.0	0.02	0.66	PVS1, PM2	-	no expert panel review available), ClinVar: 2 submissions: 1x P, 1x LP	LP
206	NM_007294.4(*BRCA1*):c.5503C>T p.(Arg1835*)	0.000065 (SAS)	-	37.0	0.02	0.55	(not applicable)	class 5 (truncating variant, non-functional protein)[3,e]	https://brcaexchange.org/variants?search=Arg1835Ter	P
251	NM_005373.3(*MPL*):c.1653+1del p.?	0.000053 (NFE)	-	-	0.98	-	PVS1, PM2	-	(no expert panel review available), ClinVar: 5 submissions: 5x P	LP
259	NM_004260.4(*RECQL4*):c.2464-1G>C p.?	0.000011 (NFE)	-	32.0	0.98	0.04	PVS1, PM2, PP1	-	(no expert panel review available), ClinVar: 2 submissions: 1x P, 1x LP	LP
273	NM_004360.5(*CDH1*):c.2200A>T p.(Arg734*)	(absent)	-	42.0	0.33	0.51	(not applicable)	ACMG/AMP criteria can be applied, as specified[5]	(no expert panel review available), ClinVar: 1 submission: 1x P	LP
293	NM_006767.4(*LZTR1*):c.2062C>T p.(Arg688Cys)	0.000124 (AFR)	0.904	28.0	0.00	0.24	PM1, PM2, PP2, PP3	-	(no expert panel review available), ClinVar: 2 submissions: 2x VUS	LP
307	NM_007194.4(*CHEK2*):c.349A>G p.(Arg117Gly)	0.000203 (NFE)	0.930	26.0	0.00	0.54	(not applicable)	class 4 (functionally relevant)[3]	https://databases.lovd.nl/shared/variants/0000148824#00024043; ClinVar: 37 submissions:9x P, 28x LP	LP
328	NM_002485.5(*NBN*):c.657_661del p.?	0.000388 (NFE)	-	27.6	0.00	-	PVS1, PS3, PM3	-	https://www.omim.org/allelicVariants/602667; ClinVar: 43 submissions: 30x P, 1x VUS	P
333	NM_016038.4(*SBDS*):c.258+2T>C p.?	0.009576 (NFE)	-	35.0	0.93	0.66	PVS1, PS3, PS4	-	(no expert panel review available), ClinVar: 50 submissions: 45x P, 3x LP, 1x risk factor, 1x not provided	P
377	NM_033084.6(*FANCD2*):c.3289C>T p.(Arg1097*)	(absent)	-	35.0	0.01	0.66	PVS1, PM2	-	(no expert panel review available); ClinVar: 1 submission: 1x P	LP

[a] Highest MAF observed in a subpopulation of gnomAD (v.2.1.1, exomes): non-Finnish European (NFE), African/African American (AFR), South Asian (SAS).

[b] The following *in silico* prediction scores available from e.g. the gnomAD and or ensembl websites, were considered for ACMG/AMP the PP3/BP1 rules, to assess the impact of the variant on gene function and splicing: The REVEL metascore is an ensembl method for predicting the pathogenicity of missense variants based on a combination of scores from 13 individual tools: MutPred, FATHMM v2.3, VEST 3.0, PolyPhen-2, SIFT, PROVEAN, MutationAssessor, MutationTaster, LRT, GERP++, SiPhy, phyloP, and phastCons[19] and levels >0.75 indicate a pathogenic effect. SpliceAI uses deep neural networks to predict splicing events. The score can range from 0 to 1, when scores can be interpreted as the probability of the variant being splice-altering; BayesDel (no AF)[20] is a deleteriousness meta-score, ranging from -1.29334 to 0.75731. The higher the score, the more likely the variant is pathogenic. In addition, the Combined Annotation Dependent Depletion (CADD) score was used to interpret the deleteriousness of single nucleotide and insertion/deletion variants [21] (https://cadd.gs.washington.edu/).

[c] ACMG/AMP criteria[2] met by the respective variant.

[d] All variants were evaluated independently by two investigators; the final conclusion/classification regarding the ACMG/AMP criteria and/or specific refinements is given here as likely pathogenic (LP) or pathogenic (P). Expert panel reviews were considered when available in the databases, e.g. ClinGen, OMIM, etc. (individual links are provided above); variant related ClinVar submissions were also listed above, when no review was available.

[e] Beside the common criteria gene specific criteria recommended recently by ClinGen for *BRCA1* variants (ClinGen ENIGMA *BRCA1* and *BRCA2* Expert Panel Specifications to the ACMG/AMP Variant Interpretation Guidelines for *BRCA1* Version 1.0.0) also indicate a damaging effect: the applicable prior was 0.99(https://hci-priors.hci.utah.edu/PRIORS/BRCA/viewer.php?gene=BRCA1); BayesDel (no AF)>0.18 (0.55; strong deleterious) for this variant.

Supplementary References

1. Gail MH, Lubin JH, Rubinstein LV. Likelihood Calculations for Matched Case-Control Studies and Survival Studies with Tied Death Times. *Biometrika* 1981; **68**(3)**:** 703-707.

2. Richards S, Aziz N, Bale S, Bick D, Das S, Gastier-Foster J*, et al.* Standards and guidelines for the interpretation of sequence variants: a joint consensus recommendation of the American College of Medical Genetics and Genomics and the Association for Molecular Pathology. *Genet Med* 2015 May; **17**(5)**:** 405-424.

3. Wappenschmidt B, Hauke J, Faust U, Niederacher D, Wiesmüller L, Schmidt G*, et al.* Criteria of the German Consortium for Hereditary Breast and Ovarian Cancer for the Classification of Germline Sequence Variants in Risk Genes for Hereditary Breast and Ovarian Cancer. *Geburtshilfe und Frauenheilkunde* 2020 Apr; **80**(4)**:** 410-429.

4. Fortuno C, Richardson M, Pesaran T, Yussuf A, Horton C, James PA*, et al.* CHEK2 is not a Li-Fraumeni syndrome gene: time to update public resources. *J Med Genet* 2023 Nov 27; **60**(12)**:** 1215-1217.

5. Lee K, Krempely K, Roberts ME, Anderson MJ, Carneiro F, Chao E*, et al.* Specifications of the ACMG/AMP variant curation guidelines for the analysis of germline CDH1 sequence variants. *Hum Mutat* 2018 Nov; **39**(11)**:** 1553-1568.

6. Mester JL, Ghosh R, Pesaran T, Huether R, Karam R, Hruska KS*, et al.* Gene-specific criteria for PTEN variant curation: Recommendations from the ClinGen PTEN Expert Panel. *Hum Mutat* 2018 Nov; **39**(11)**:** 1581-1592.

7. Luo X, Feurstein S, Mohan S, Porter CC, Jackson SA, Keel S*, et al.* ClinGen Myeloid Malignancy Variant Curation Expert Panel recommendations for germline RUNX1 variants. *Blood Adv* 2019 Oct 22; **3**(20)**:** 2962-2979.

8. Fortuno C, Lee K, Olivier M, Pesaran T, Mai PL, de Andrade KC*, et al.* Specifications of the ACMG/AMP variant interpretation guidelines for germline TP53 variants. *Hum Mutat* 2021 Mar; **42**(3)**:** 223-236.

9. Thompson BA, Spurdle AB, Plazzer JP, Greenblatt MS, Akagi K, Al-Mulla F*, et al.* Application of a 5-tiered scheme for standardized classification of 2,360 unique mismatch repair gene variants in the InSiGHT locus-specific database. *Nat Genet* 2014 Feb; **46**(2)**:** 107-115.

10. Brinkmann J, Lissewski C, Pinna V, Vial Y, Pantaleoni F, Lepri F*, et al.* The clinical significance of A2ML1 variants in Noonan syndrome has to be reconsidered. *European Journal of Human Genetics* 2021 2021/03/01; **29**(3)**:** 524-527.

11. Arber DA, Orazi A, Hasserjian R, Thiele J, Borowitz MJ, Le Beau MM*, et al.* The 2016 revision to the World Health Organization classification of myeloid neoplasms and acute leukemia. *Blood* 2016 May 19; **127**(20)**:** 2391-2405.

12. Andrés-Zayas C, Suárez-González J, Rodríguez-Macías G, Dorado N, Osorio S, Font P*, et al.* Clinical utility of targeted next-generation sequencing for the diagnosis of myeloid neoplasms with germline predisposition. *Mol Oncol* 2021 Sep; **15**(9)**:** 2273-2284.

13. Prins D, González Arias C, Klampfl T, Grinfeld J, Green AR. Mutant Calreticulin in the Myeloproliferative Neoplasms. *Hemasphere* 2020 Feb; **4**(1)**:** e333.

14. Byrjalsen A, Diets IJ, Bakhuizen J, Hansen TVO, Schmiegelow K, Gerdes AM*, et al.* Selection criteria for assembling a pediatric cancer predisposition syndrome gene panel. *Fam Cancer* 2021 Oct; **20**(4)**:** 279-287.

15. Southey MC, Goldgar DE, Winqvist R, Pylkäs K, Couch F, Tischkowitz M*, et al.* *PALB2*, *CHEK2* and *ATM* rare variants and cancer risk: data from COGS. *Journal of Medical Genetics* 2016; **53**(12)**:** 800-811.

16. Dorling L, Carvalho S, Allen J, Parsons MT, Fortuno C, González-Neira A*, et al.* Breast cancer risks associated with missense variants in breast cancer susceptibility genes. *Genome Medicine* 2022 2022/05/18; **14**(1)**:** 51.

17. de Andrade KC, Frone MN, Wegman-Ostrosky T, Khincha PP, Kim J, Amadou A*, et al.* Variable population prevalence estimates of germline TP53 variants: A gnomAD-based analysis. *Hum Mutat* 2019 Jan; **40**(1)**:** 97-105.

18. Haigis KM. KRAS Alleles: The Devil Is in the Detail. *Trends Cancer* 2017 Oct; **3**(10)**:** 686-697.

19. Ioannidis NM, Rothstein JH, Pejaver V, Middha S, McDonnell SK, Baheti S*, et al.* REVEL: An Ensemble Method for Predicting the Pathogenicity of Rare Missense Variants. *Am J Hum Genet* 2016 Oct 6; **99**(4)**:** 877-885.

20. Pejaver V, Byrne AB, Feng BJ, Pagel KA, Mooney SD, Karchin R*, et al.* Calibration of computational tools for missense variant pathogenicity classification and ClinGen recommendations for PP3/BP4 criteria. *Am J Hum Genet* 2022 Dec 1; **109**(12)**:** 2163-2177.

21. Rentzsch P, Schubach M, Shendure J, Kircher M. CADD-Splice—improving genome-wide variant effect prediction using deep learning-derived splice scores. *Genome Medicine* 2021 2021/02/22; **13**(1)**:** 31.

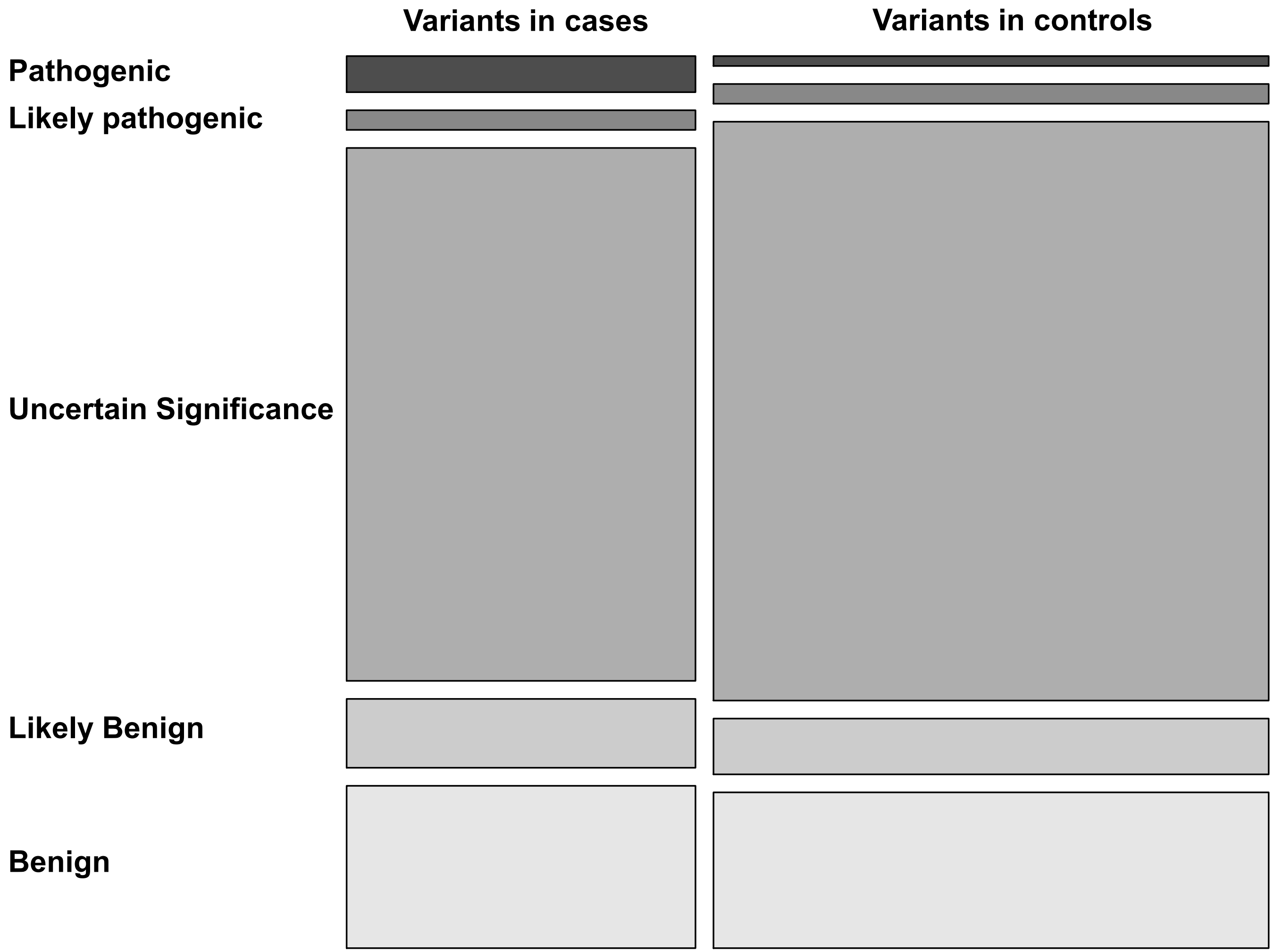

Variant classification	Amount of variants in patients with ALL+SMN, n=248(%)	Amount of variants in patients with ALL, n=395(%)	$P(X^2)$
Pathogenic	11(4.44%)	5(1.27%)	
Likely pathogenic	6(2.42%)	9(2.28%)	
Uncertain significance	161(64.92%)	279(70.63%)	0.112
Likely benign	21(8.47%)	27(6.84%)	
Benign	49(19.76%)	75(18.99%)	

Supplementary Figure 1. Distribution of classified variants by case-control status of the carrier. The frequency distribution was analyzed using Pearson's Chi-squared test: X^2 = 7.4816, df = 4, p-value = 0.1125.
